# The effect of S53P4-based borosilicate glasses and glass dissolution products on the osteogenic commitment of human adipose stem cells

**DOI:** 10.1371/journal.pone.0202740

**Published:** 2018-08-28

**Authors:** Miina Ojansivu, Ayush Mishra, Sari Vanhatupa, Miia Juntunen, Antonina Larionova, Jonathan Massera, Susanna Miettinen

**Affiliations:** 1 Faculty of Medicine and Life Sciences and BioMediTech Institute, University of Tampere, Tampere, Finland; 2 Science Center, Tampere University Hospital, Tampere, Finland; 3 Faculty of Biomedical Science and Engineering and BioMediTech Institute, Tampere University of Technology, Tampere, Finland; Friedrich-Alexander-Universitat Erlangen-Nurnberg, GERMANY

## Abstract

Despite the good performance of silicate bioactive glasses in bone regeneration, there is considerable potential to enhance their properties by chemical modifications. In this study, S53P4-based borosilicate glasses were synthesized and their dissolution profile was studied in simulated body fluid by assessing pH change, ion release and conversion to hydroxyapatite. The viability, proliferation, attachment, osteogenesis and endothelial marker expression of human adipose stem cells (hASCs) was evaluated upon direct culture on glass discs and in the extract medium. This is the first study evaluating cell behavior in response to borosilicate glasses based on S53P4 (commercially available as BonAlive^®^). Replacing silicate with borate in S53P4 increased the glass reactivity. Despite the good viability of hASCs under all conditions, direct culture of cells on borosilicate discs and in undiluted extract medium reduced cell proliferation. This was accompanied with changes in cell morphology. Regarding osteogenic commitment, alkaline phosphatase activity was significantly reduced by the borosilicate glass discs and extracts, whereas the expression of osteogenic markers *RUNX2a*, *OSTERIX*, *DLX5* and *OSTEOPONTIN* was upregulated. There was also a borosilicate glass-induced increase in osteocalcin protein production. Moreover, osteogenic supplements containing borosilicate extracts significantly increased the mineral production in comparison to the osteogenic medium control. Interestingly, borosilicate glasses stimulated the expression of endothelial markers *vWF* and *PECAM-1*. To conclude, our results reveal that despite reducing hASC proliferation, S53P4-based borosilicate glasses and their dissolution products stimulate osteogenic commitment and upregulate endothelial markers, thus supporting their further evaluation for regenerative medicine.

## Introduction

Since the invention of the original bioactive glass (BaG) composition 45S5 by Larry Hench in1969[1], research on BaGs has expanded almost exponentially. Novel glass compositions, manufacturing strategies and structures, as well as new application areas, are constantly being introduced by numerous research groups. However, considering the extensive amount of studies conducted with BaGs, the high expectations regarding their clinical translation have not been entirely fulfilled. Some commercial BaG products for clinical use are in the market, but they are mainly simple particulate formulations intended for bone fillers in dental and orthopedic applications, and their compositions are mostly 45S5 or a similar silicate, S53P4[2]. Moreover, despite the generally good performance of these glasses, they have been observed to remain in the body even 14 years post-operation[3], which clearly necessitates a re-evaluation of the glass compositions to facilitate a faster degradation rate.

Chemical modifications of glass compositions offer an easily exploitable toolkit to adjust the glass properties toward a desired outcome [4]. For example, boron addition to silicate BaGs has been found to disrupt the silica network and increase glass dissolution rate in aqueous medium[5,6]. Consequently, the replacement of silica with borate in compositions 45S5 and 13–93 leads to a greatly increased conversion rate of glass to hydroxyapatite (HA) in simulated body fluid (SBF) and in dilute phosphate solutions[7–9]. In the case of glass 13–93, complete replacement of silicate with borate increased the HA conversion rate of porous scaffolds to 3–4 times that of 13–93[8]. Moreover, 45S5-based borate glass particles (150–300 μm) had almost completely converted to HA in less than four days, whereas only partial (50%) conversion of silicate 45S5 glass particles was detected even after 70 days in incubation[7].

Dietary boron is considered an essential trace element, which regulates bone metabolism and has a stimulatory effect on bone formation[10–12]. However, the effects of boron at the cellular level remain poorly understood. Ying and coworkers observed that osteogenic medium supplemented with boron (obtained from boric acid) at concentrations of 1–100 ng/mL can stimulate osteogenic differentiation of human bone marrow-derived mesenchymal stem cells (hBMSCs) in terms of alkaline phosphatase (ALP) activity, mineralization and osteogenic marker gene expression[13]. Similar observations were made with murine osteoblastic cell line MC3T3-E1[14]. However, boron concentrations of 1,000 ng/mL and above were observed to decrease cell viability and proliferation, suggesting that boron can be toxic to the cells above certain concentrations[13,14]. In line with these observations regarding boron toxicity, the ability of 45S5- and 13-93-based borate and borosilicate glasses to support cell viability and proliferation has been generally poorer than those of the corresponding silicate glasses, both in direct culture and in glass-ion conditioned media[7,15–18]. As expected, this inhibitory effect seems to correlate with the borate content of the glass.

Since bone formation is known to be accompanied with reduction in cell amount[19], it is still possible that borosilicate BaGs have beneficial effects on osteogenic commitment of precursor cells. Regarding this issue, it has been demonstrated that glass-induced ALP activity is downregulated by replacement of silicate with borate in 13–93 glass[15–17]. However, ALP activity alone is not considered a reliable indicator of osteogenic commitment[20–22], and therefore to be able to better understand the cellular responses to borosilicate glasses, a more thorough evaluation of borosilicate BaG-induced differentiation is required. Moreover, considering their favorable dissolution behavior and promising induction of bone formation *in vivo*[23–25], borosilicate BaGs still possess high potential for clinical translation. This goal also necessitates more detailed knowledge about cell response *in vitro*.

The aim of this study was to evaluate the impact of SiO_2_ substitution by B_2_O_3_ in S53P4 glass composition on the glass dissolution profile and the behavior of human adipose stem cells (hASCs). To the best of our knowledge, this is the first time that S53P4-based borosilicate glasses have been evaluated. S53P4, commercially available as BonAlive^®^, has been widely utilized in clinics and, combined with autologous hASCs, has been successfully used in bone tissue engineering based clinical treatments of frontal sinus defects[26]. However, similarly to other silicate glasses, it suffers from slow degradation rate and retention in the body even several years post-operation[3]. Here we evaluate the effect of substituting silicate with borate in S53P4 on the glass dissolution behavior. Moreover, we demonstrate the effect of S53P4-based borosilicate glasses on hASC viability, proliferation and morphology, as well as early and late osteogenic commitment, both upon direct culture on BaG discs and in glass ion-conditioned extract media. The extract media were prepared from BaG granules, as outlined in our previous work with silicate glasses[22]. Finally, since bone tissue is highly dependent on proper vascularization, the expression of endothelial marker genes in response to these glasses was evaluated. It has previously been shown that 45S5-based borosilicate glass dissolution products can induce human umbilical vein endothelial cell (HUVEC) tubule formation and pro-angiogenic cytokine production[27], but the endothelial response of mesenchymal stem cells (MSCs) to borosilicate glasses is not known.

## Materials and methods

### Ethical statement

This study was conducted in accordance with the ethical approval granted by the Ethics Committee of Pirkanmaa Hospital District, Tampere, Finland (R15161). The hASCs were isolated from surgically removed subcutaneous adipose tissue pieces obtained from the Department of Plastic Surgery, Tampere University Hospital. There were five female donors of age 54 ± 11 years. Written informed consent was obtained from all donors for the utilization of adipose tissue samples for research purposes.

### Bioactive glass synthesis, characterization and pretreatment prior to cell culture

Bioactive glasses were melted from batches containing mixtures of sand (99.4% pure SiO_2_) and analytical grade Na_2_CO_3_, H_3_BO_3_, CaCO_3_ and CaHPO_4_·2H_2_O. The nominal oxide compositions of the experimental glasses in mol-% can be represented by the formula: (53.85-x)SiO_2_-xB_2_O_3_-22.66Na_2_O-1.72P_2_O_5_-21.77CaO, where x varies from 0 to 26.93. The nominal compositions are also shown in Table 1. The glasses were coded as follows: S53P4 (x = 0), B25 (x = 13.46) and B50 (x = 26.93). The glasses were melted in air in a platinum crucible at temperatures ranging from 1,250 to 1,400 °C for 3 h, depending on the boron content. After casting in a pre-heated cylindrical (Ø = 10 or 14 mm) graphite mold, the glasses were annealed overnight at 40 °C below their respective glass transition temperatures T_g_, and then allowed to cool slowly to room temperature in the annealing furnace. The obtained rods were either crushed into particles or sliced into discs (h = 2 mm), which were grounded and polished.

**Table 1:** The nominal compositions of the BaGs used in this study.

	mol-%				
	*Na*_*2*_*O*	*CaO*	*P*_*2*_*O*_*5*_	*SiO*_*2*_	*B*_*2*_*O*_*3*_
**S53P4**	22.66	21.77	1.72	53.85	0
**B25**	22.66	21.77	1.72	40.39	13.46
**B50**	22.66	21.77	1.72	29.93	29.93

To analyze glass dissolution, glass particles with grain sizes of 250–500 μm were immersed in 50 mL SBF for 6, 24, 48, 72, and 168 h at 37 °C in an incubating shaker (INFORS Multitron II; Infors HT, Bottmingen, Switzerland). In the shaker, an orbital speed of 100 rpm was chosen to give laminar flow mixing of the solutions, without inducing particle movement. SBF was prepared using the protocol developed by Kokubo et al.[28]. The mass of the sample immersed in solution was adjusted to give a constant surface area (SA) to volume ratio. The calculations were made using the glass density, with the assumption that the average particle diameter was 187.5 μm. At each time point, four parallel samples of each glass composition were studied. The change in the solution’s pH was recorded at each immersion time-point and compared to a blank sample containing only SBF. After testing, the particles were washed with acetone and dried. A portion of the particles was embedded in resin and polished to reveal the particles’ cross section. The same technique was applied to the glass discs after 21d under cell culture conditions. The composition and structure of the glass particles and discs were analyzed by scanning electron microscopy/energy-dispersive X-ray analysis (SEM/EDX; Leo 1530 Gemini (Zeiss, Oberkochen, Germany) and EDXA UltraDry (Thermo Fisher Scientific, Waltham, MA, USA)). For the ion concentration analysis, 5 mL SBF was collected, diluted 10 times and analyzed using inductively coupled plasma optical emission spectrometry (ICP-OES; Agilent 5100 ICP-OES; Santa Clara, California, USA). The elements analyzed by ICP-OES were Na (λ = 589.592 nm), Ca (λ = 393.366 nm), P (λ = 253.56nm), B (λ = 249.772 nm) and Si (λ = 251.611 nm).

Prior to use in cell culture experiments, glasses were disinfected by incubating them twice for 10 min in 70% ethanol, followed by air-drying in the cell culture laminar hood. Glass discs were incubated overnight in basic cell culture medium prior to cell seeding.

Glass ion-conditioned extract was prepared from B25 glass granules (500–1,000 μm) as described elsewhere [20]. Undiluted extract (B1), as well as 1:10 (B2) and 1:100 (B3) dilutions, prepared using cell culture medium, were analyzed. The results of the ICP-OES are shown in Table 2.

**Table 2:** Ion concentrations within BaG-free DMEM/F-12 and BaG extract media. The extract media were prepared from B25 glass granules. In addition to undiluted extract (B1), dilutions of 1:10 (B2) and 1:100 (B3) were evaluated.

	Na (mg/L)	Ca (mg/L)	P (mg/L)	Si (mg/L)	B (μg/L)
**DMEM/F-12**	3,160	42.4	28.9	<1	21.3
**B1**	3,380	121	14.4	54.3	50,500
**B2**	3,270	49.9	27.8	5.85	5,450
**B3**	3,190	42.7	28.6	<1	660

### Isolation and culture of human adipose stem cells

Human ASCs were isolated with a mechanical and enzymatic procedure, as previously described[29,30]. The isolated hASCs were maintained in T-75 polystyrene flasks (Nunc, Thermo Fisher Scientific) in DMEM/F-12 (Life Technologies, Thermo Fisher Scientific) supplemented with 5% human serum (HS; Biowest, Nuaillé, France), 1% L-glutamine (GlutaMAX I, Life Technologies, Thermo Fisher Scientific) and 1% antibiotics (100 U/mL penicillin and 0.1 mg/mL streptomycin; BioWittaker, Lonza, Basel, Switzerland). This medium composition is referred to as basic medium (BM). Osteogenic medium (OM; BM supplemented with 250 μM L-ascorbic acid 2-phosphate, 10 mM β-glycerophosphate and 5 nM dexamethasone; all from Sigma-Aldrich, St. Louis, MO, USA) was also prepared. The mesenchymal origin of the hASCs was verified by surface marker expression analysis at passage 1, conducted with flow cytometry (FACSAria, Becton, Dickinson and Company, Erembodegem, Belgium)[31]. The hASCs strongly expressed surface markers CD73, CD90 and CD105, whereas the expression of CD3, CD11a, CD14, CD19, CD45, CD80, CD86, HLA-DR, CD34 and CD54 was either low or moderate (Table 3), thus verifying their mesenchymal origin. Passages 2–5 were used for experimentation.

**Table 3:** The surface marker expression of undifferentiated hASCs at passage 1, cultured in BM.

Antigen	Surface protein	Expression	Fluorophore	Manufacturer
CD3	T-cell surface glycoprotein	0.4 ± 0.4	phycoerythrin (PE)	BD Biosciences, Franklin Lakes, NJ, USA
CD11a	Lymphocyte function-associated antigen 1	1.0 ± 0.7	allophycocyanin (APC)	R&D Systems, Minneapolis, MN, USA
CD14	Serum lipopolysaccharide binding protein	0.8 ± 0.3	phycoerythrincyanine (PECy7)	BD Biosciences
CD19	B lymphocyte-lineage differentiation antigen	0.5 ± 0.4	PECy7	BD Biosciences
CD34	Sialomucin-like adhesion molecule	19.6 ± 26.5	APC	Immunotools, Friesoythe, Germany
CD45	Leukocyte common antigen	1.7 ± 0.6	APC	BD Biosciences
CD54	Intercellular adhesion molecule 1	10.7 ± 5.9	fluorescein icothiocyanate (FITC)	BD Biosciences
CD73	Ecto-5'-nucleotidase	95.4 ± 4.7	PE	BD Biosciences
CD80	B-lymphocyte activation antigen B7	0.5 ± 0.3	PE	R&D Systems
CD86	B-lymphocyte activation antigen B7-2	0.5 ± 0.3	PE	R&D Systems
CD90	Thy-1 (T cell surface glycoprotein)	98.9 ± 1.2	APC	BD Biosciences
CD105	SH-2, endoglin	96.0 ± 5.5	PE	R&D Systems
HLA-DR	Major histocompatibility class II antigens	0.5 ± 0.3	PE	Immunotools

Cells were plated on discs at 5,500 cells/cm^2^ and in extracts at 1,100 cells/cm^2^. Cultures were grown on polystyrene for controls. Cells were plated in BM and the medium was changed the following day (day 0). In the BaG disc experiment, half of the wells received BM and half OM. In case of the extract experiment, both BM- and OM-based extracts were used in addition to the control BM and OM. The media were changed twice weekly during the experiments.

### Assessment of cell viability and proliferation

Cell viability on BaG discs and in BaG extracts was analyzed with Live/Dead staining (Invitrogen, Thermo Fisher Scientific) as previously described[22]. Briefly, cells were incubated in working solution containing 0.25 μM EthD-1 and 0.5 μM Calcein-AM for 20 min at room temperature. This was followed by immediate imaging (IX51, Olympus, Tokyo, Japan, equipped with a fluorescence unit and a camera DP30BW, Olympus).

Cell proliferation was assessed after 7 and 14 days of culture using CyQUANT Cell Proliferation Assay (Invitrogen, Thermo Fisher Scientific), according to the manufacturer’s protocol. Samples were lysed in 0.1% TritonX-100 lysis buffer (Sigma-Aldrich), followed by freezing and thawing once at -70 °C. Three parallel 20 μl replicates of each lysate were pipetted to a 96-well plate (Nunc) and mixed with 180 μl working solution. The fluorescence at 480/520 nm was measured using plate reader (Victor 1420 Multilabel counter,Wallac, Turku, Finland).

### Alkaline phosphatase activity and mineralization analysis

Alkaline phosphatase activity, an indicator of early osteogenic commitment, was quantitatively evaluated after 7 and 14 days of culture as described previously[22]. Briefly, 20 μl of cell lysate (the same as used for the proliferation assay) was combined with 90 μl working solution (alkaline buffer solution and phosphatase substrate mixed in equal amounts; both from Sigma-Aldrich) in a 96-well plate and incubated for 15 min at +37°C. The reaction was stopped by adding 50 μl 1 M NaOH (Sigma-Aldrich) and the absorbance was measured at 405 nm with Victor 1420 Multilabel counter (Wallac). To assess the late osteogenesis of hASCs cultured in glass extracts, Alizarin red S staining of calcium phosphate (CaP) mineral was conducted after 19 days of culture. Cells cultured on BaG discs could not be characterized with Alizarin red S due to the heavy background staining of the discs. In brief, cells were fixed with 4% paraformaldehyde (PFA) and stained with 2% Alizarin red S (pH 4.1–4.3; Sigma-Aldrich) solution for 10 min at room temperature. After washes, the samples were imaged. Finally, the dye was extracted with 100 mM cetylpyridinium chloride (Sigma-Aldrich) and the result was quantified by measuring the absorbances at 544 nm (Victor 1420 Multilabel counter).

### Quantitative real-time PCR

The relative expression of osteogenic marker genes *RUNX2a*, *OSTERIX*, *DLX5*, *OSTEOPONTIN* and *OSTEOCALCIN*, as well as the expression of endothelial markers *von Willebrand factor (vWF)* and *PECAM-1*, was studied after 7 and 14 days of culture by quantitative real-time reverse transcription polymerase chain reaction (qRT-PCR) as described previously[22]. Housekeeping gene human acidic ribosomal phosphoprotein P0 (RPLP0) was used to normalize the data. A mathematical model described by Pfaffl was used to calculate the relative expressions[32]. For *PECAM-1*, QuantiTect Primer Assay was utilized (Qiagen, Hilden, Germany). The primer sequences of the other genes (Oligomer Oy, Helsinki, Finland) and the mRNAs accession numbers are presented in Table 4.

**Table 4:** The primer sequences and the accession numbers of the mRNAs studied with qRT-PCR.

Name	5’-Sequence-3’	Product size (bp)	Accession Number
***DLX5***	forward ACCATCCGTCTCAGGAATCG reverse CCCCCGTAGGGCTGTAGTAGT	75	NM_005221.5
***OSTEOCALCIN***	forward AGCAAAGGTGCAGCCTTTGT reverse GCGCCTGGGTCTCTTCACT	63	NM_199173.5
***OSTEOPONTIN***	forward GCCGACCAAGGAAAACTCACT reverse GGCACAGGTGATGCCTAGGA	71	J04765
***OSTERIX***	forward TGAGCTGGAGCGTCATGTG reverse TCGGGTAAAGCGCTTGGA	79	AF477981
***RPLP0***	forward AATCTCCAGGGGCACCATT reverse CGCTGGCTCCCACTTTGT	70	NM_001002
***RUNX2a***	forward CTTCATTCGCCTCACAAACAAC reverse TCCTCCTGGAGAAAGTTTGCA	62	NM_001024630.3
***vWF***	forward AGAAACGCTCCTTCTCGATTATTG reverse TGTCAAAAAATTCCCCAAGATACAC	84	NM_000552.4

### Immunocytochemical stainings

To further evaluate the late osteogenic differentiation as well as the maturation of extracellular matrix (ECM), collagen-I and osteocalcin immunocytochemical stainings were conducted, as described in detail previously[22]. Primary antibodies mouse monoclonal anti-collagen-I (dilution 1:2000) and mouse monoclonal anti-osteocalcin (dilution 1:100) (Abcam, Cambridge, UK) and secondary antibody donkey anti-mouse Alexa fluor 488 IgG (Invitrogen, Thermo Fisher Scientific; dilution 1:800) were used. Secondary antibody was applied together with actin-staining phalloidin-TRITC (Sigma-Aldrich, dilution 1:500). The nuclei were stained with DAPI (Molecular Probes, Thermo Fisher Scientific; dilution 1:2000) during the wash steps after the secondary antibody treatment.

In addition to the collagen-I and osteocalcin stainings, the structure of the actin cytoskeleton was visualized with phalloidin staining after 7 days of culture. For this, the samples were fixed, permeabilized and blocked, and the phalloidin and DAPI stainings were conducted as described in ref.[22], but with the omission of the antibody treatments. All the samples were imaged with the aforementioned microscope.

### Statistical analysis

Statistical analyses were conducted using SPSS Statistics version 22 software (IBM, Armonk, NY, USA). Quantitative data is presented as mean plus standard deviation. Kruskal-Wallis one-way analysis of variance by ranks, and Mann-Whitney post hoc test combined with Bonferroni correction, were used to evaluate the statistical significances. Statistical significance was considered to exist when the adjusted p-value was < 0.05.

## Results

### Glass dissolution in simulated body fluid

**Fig 1:**
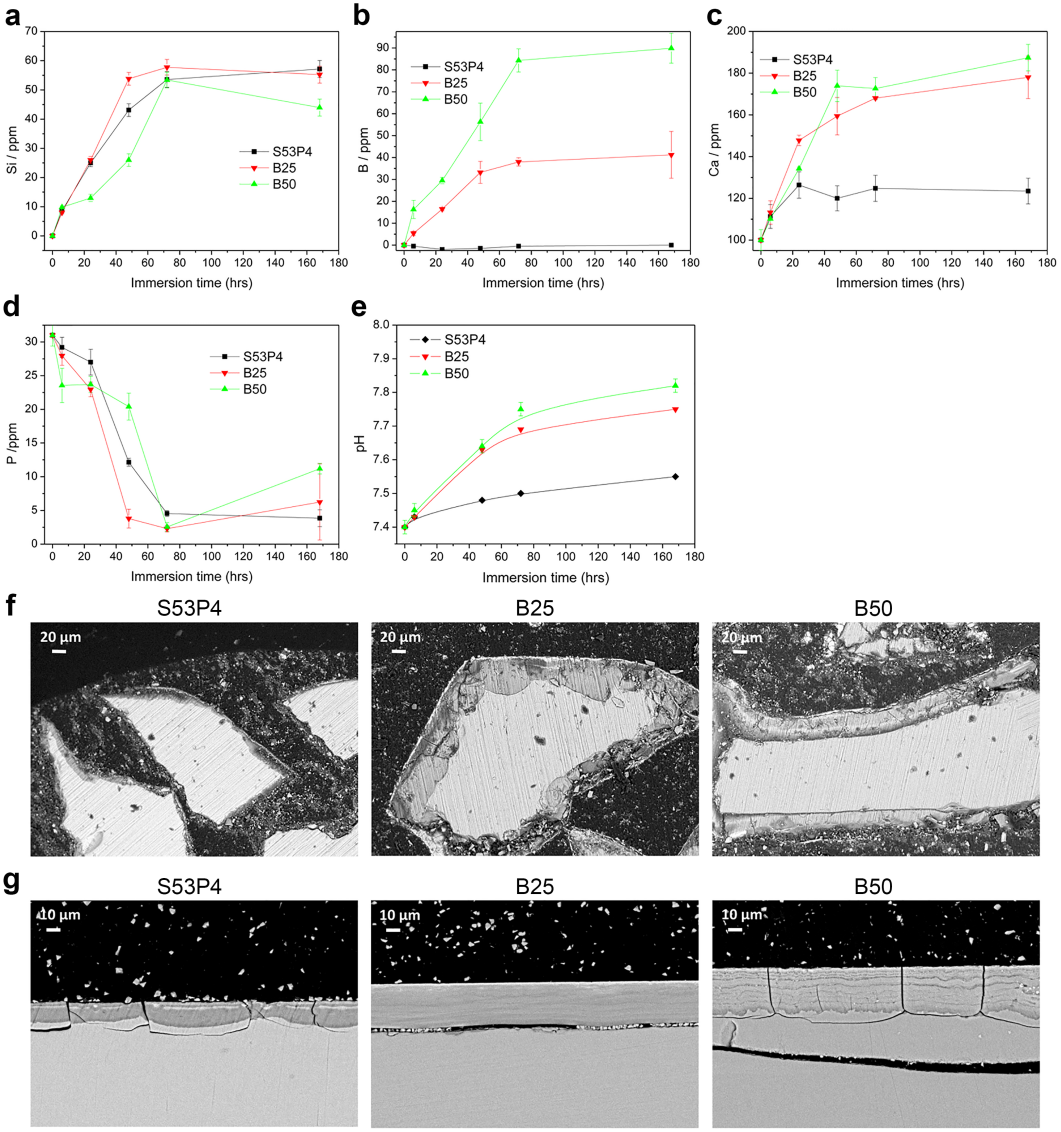
Characterization of glass dissolution and reaction layer formation. **a-d**. Si, B, Ca and P concentrations, respectively, in SBF as a function of immersion time. **e**. The pH of SBF solution as a function of immersion time. **f**. SEM images of S53P4, B25 and B50 glass particles immersed for 7d in SBF. Scale bar 20 μm. **g**. SEM images of S53P4, B25 and B50 glass disc cross-sections, after immersion for 21 days in cell culture medium. Scale bar 10 μm.

Fig 1a–1d presents the ion concentrations in the SBF solution as a function of immersion time. The concentration of Si increased with increasing immersion time for all the investigated samples (Fig 1a). However, no drastic differences in Si concentration were detected as a function of boron content. The concentration of B, on the other hand, not only increased with increasing immersion time but also with increasing boron content in the glass (Fig 1b). In case of S53P4, the concentration of Ca increased for up to 48 h of immersions and then remained constant over the rest of the immersion time (Fig 1c). In contrast to this, both boron containing glasses exhibited an increase in Ca concentration throughout the whole immersion period (Fig 1c). However, no clear differences in Ca release were observed between the B25 and B50 glasses. Lastly, the concentration of P was found to decrease with increasing immersion time, with no clear effect of the boron content on its release (Fig 1d). Fig 1e shows the pH change of the SBF after immersion of the glass particles for up to 336 h. As expected, an increase in immersion time led to an increase in pH. This was higher in the cases of boron-containing glasses B25 and B50.

In order to evaluate the reaction layer formation, the glasses were analyzed with SEM/EDX post-immersion. Fig 1f presents the SEM images of the glass particles after one week of SBF immersion. In Fig 1g cross-sections of glass discs after 21 days of cell culture are shown. Above the remaining layer of unreacted glass, a darker layer rich in Si and a brighter reactive top layer of CaP are clearly visible. The Si and CaP layers were found to increase in thickness with increasing boron content in the glass. The top layer on all of the glass compositions had a Ca/P atomic ratio of ~1.60–1.65, indicating HA formation.

### Cell viability and proliferation on BaG discs and in BaG extracts

**Fig 2:**
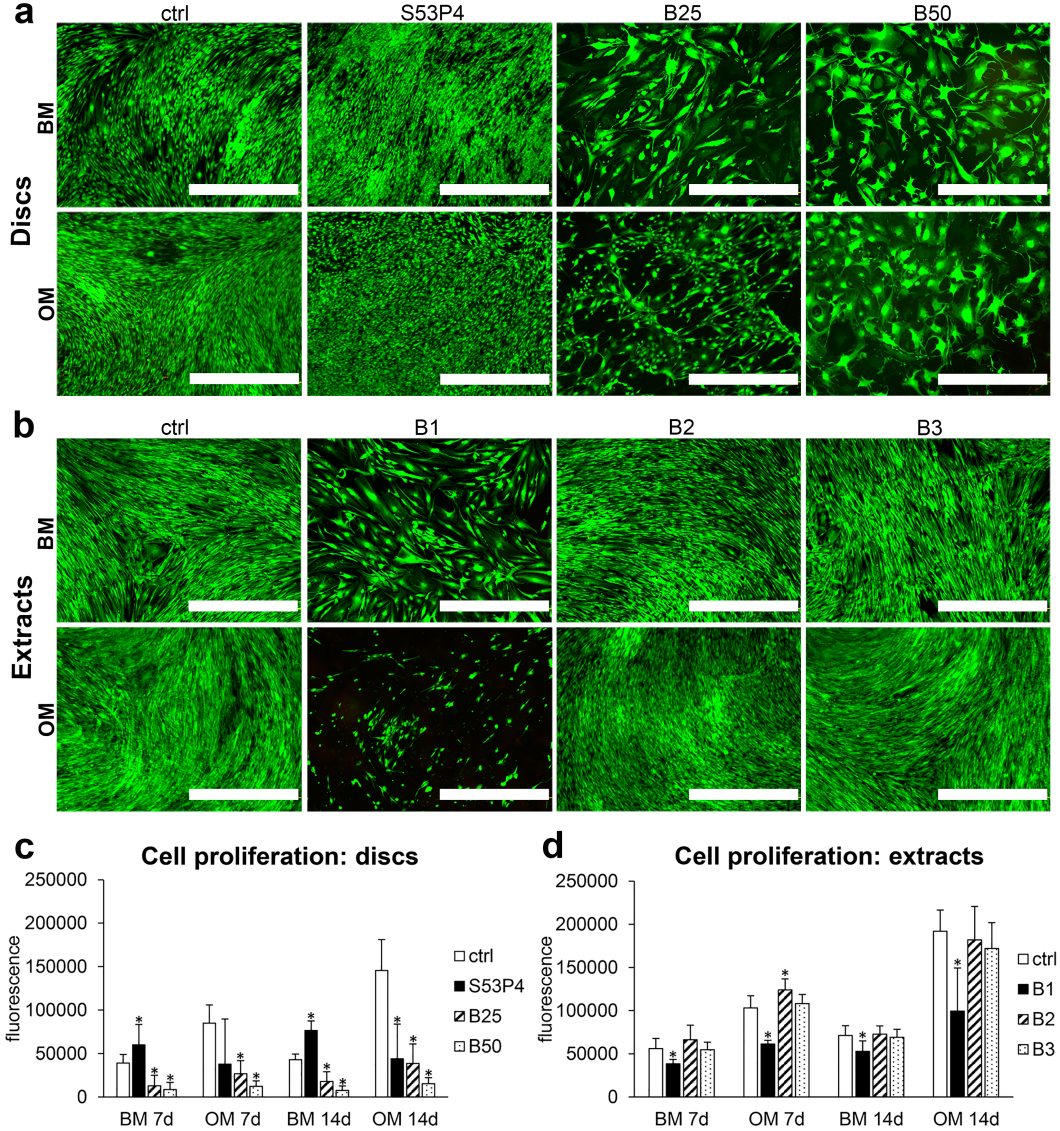
Cell viability and proliferation in response to borosilicate glasses. **a**. Cell viability on bioactive glass discs at 21d. **b**. Cell viability in borosilicate glass extracts at 19d. Viability was analyzed with Live/dead staining. Scale bars 1.0 mm. **c**. Cell proliferation on glass discs (n = 9). **d**. Cell proliferation in glass extracts (n = 12). Proliferation was analyzed with CyQUANT assay. * p<0.05 between the indicated group and the control (ctrl) group at the same time point. BM = basic medium, OM = osteogenic medium. B1 = undiluted extract, B2 = 1:10 dilution, B3 = 1:100 dilution.

As seen in Fig 2a, the viability of hASCs on all glass disc compositions remained good throughout the culture period, since no dead cells could be observed. However, it is already evident from the live/dead staining images that especially, on the boron-containing glasses (B25 and B50), there were fewer cells compared to the control and S53P4 reference glass. This observation was verified with quantitative proliferation analysis, which showed a significantly lower cell amount on B25 and B50, compared to the control at both time points and in both BM and OM (Fig 2c). In the case of S53P4, proliferation significantly increased in BM. By contrast, hASC proliferation decreased in OM. Regarding the extracts, no dead cells could be detected under either condition (Fig 2b), but undiluted extract (B1) significantly attenuated proliferation (Fig 2d). However, diluted extracts did not affect proliferation in comparison to the control conditions.

### The effect of BaG disc and extract cultures on hASC morphology

**Fig 3:**
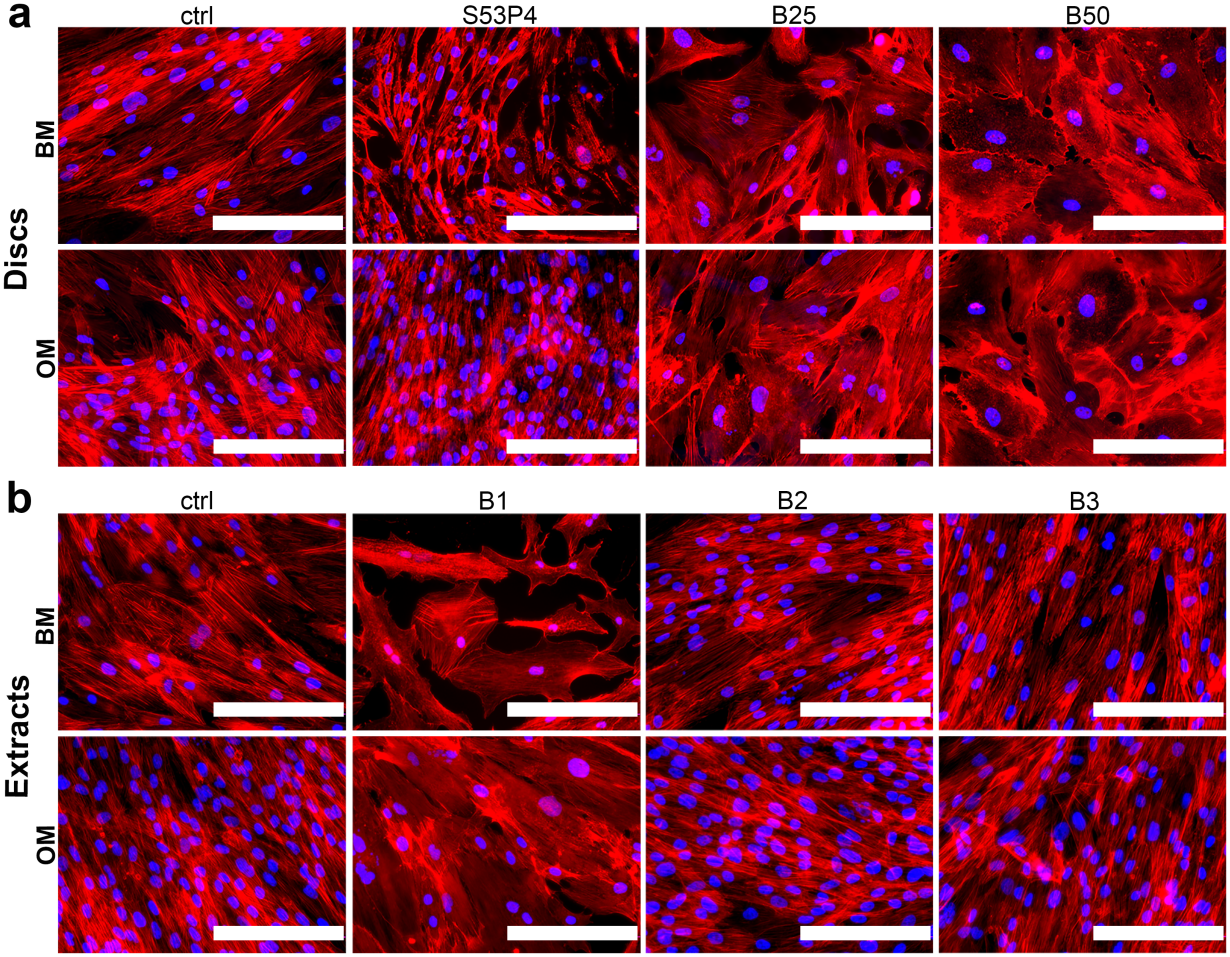
Cell morphology in response to borosilicate glasses. **a**. Phalloidin staining (red) of actin cytoskeleton of hASCs cultured on glass discs for 7d. **b**. Phalloidin staining (red) of actin cytoskeleton of hASCs cultured in glass extracts for 7d. Nuclei were stained blue with DAPI. Scale bars 200 μm. BM = basic medium, OM = osteogenic medium. B1 = undiluted extract, B2 = 1:10 dilution, B3 = 1:100 dilution.

As evidenced already by the live/dead staining (Fig 2a and 2b), the cell morphology was affected by the borosilicate glasses which prompted us to further evaluate the morphology by staining the actin cytoskeleton (Fig 3). Regardless of the presence of osteogenic supplements, the hASCs adopted an enlarged morphology when cultured on B25 and B50 glasses when compared to the typical spindle-shaped MSC appearance in control and on S53P4 glass (Fig 3a). Moreover, the actin cytoskeleton was partially disrupted and in some cells it was clearly evident only on the periphery of the cells. However, despite these morphological changes, the cells grew in tight contact with each other and formed a confluent cell layer on the borosilicate glasses. Interestingly, a very similar morphological appearance was observed when the cells were cultured in undiluted BaG extract (Fig 3b). Diluted extracts, on the other hand, did not cause this enlarged morphology.

### Early osteogenic commitment induced by the BaG discs and extracts

**Fig 4:**
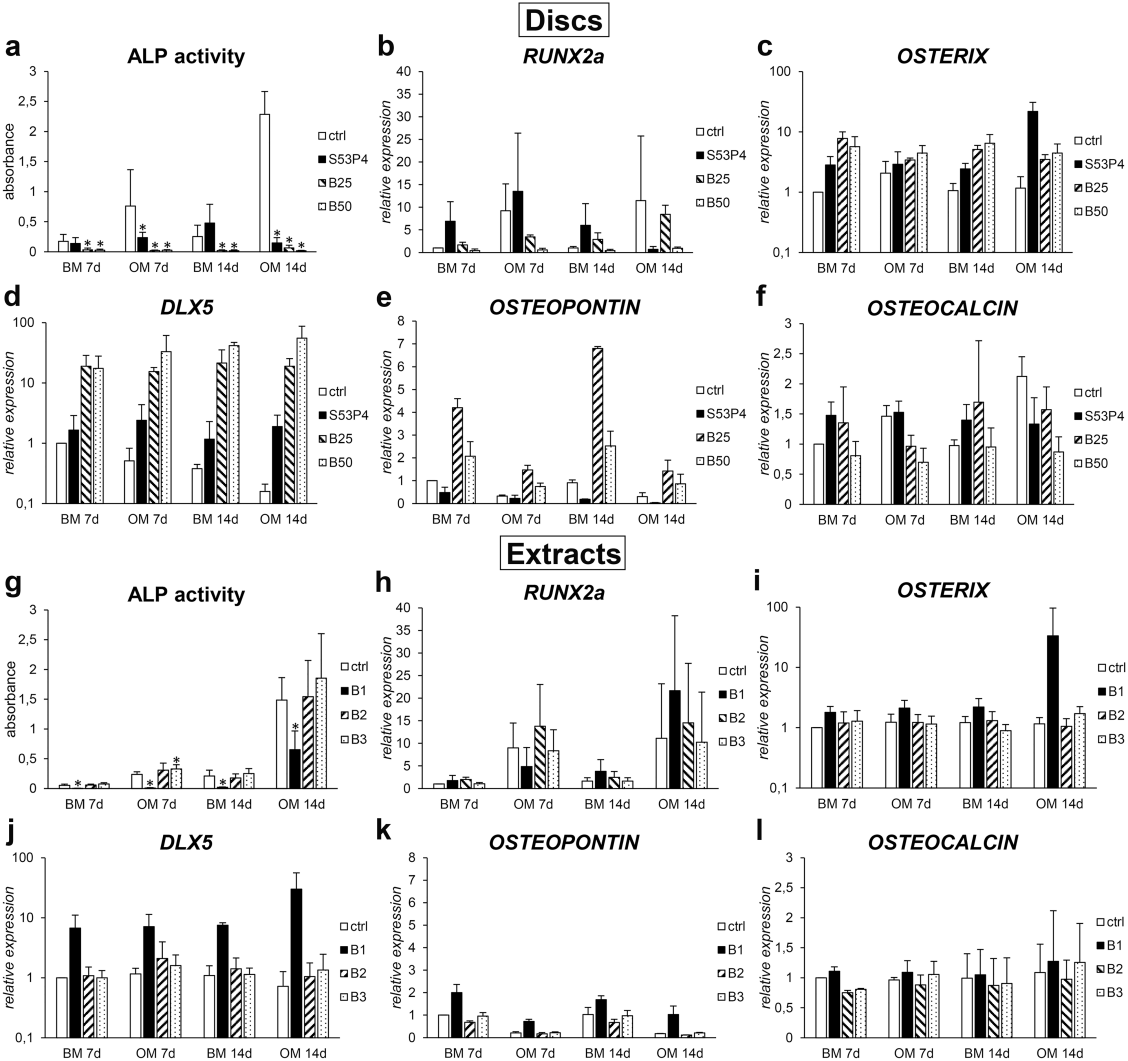
Borosilicate glass-induced early osteogenic commitment of hASCs. **a**. ALP activity (n = 9) and expression of osteogenic marker genes *RUNX2a* (**b**), *OSTERIX* (**c**), *DLX5* (**d**), *OSTEOPONTIN* (**e**) and *OSTEOCALCIN* (f) (n = 2–4) in hASCs cultured on glass discs. **g**. ALP activity (n = 12) and the expression of osteogenic marker genes *RUNX2a* (**h**), *OSTERIX* (**i**), *DLX5* (**j**), *OSTEOPONTIN* (**k**) and *OSTEOCALCIN* (**l**) (n = 3–4) in hASCs cultured in glass extracts. * p<0.05 between the indicated group and the control (ctrl) group at the same time point. BM = basic medium, OM = osteogenic medium. B1 = undiluted extract, B2 = 1:10 dilution, B3 = 1:100 dilution.

To determine the early osteogenic commitment of hASCs in response to culture on BaG discs and in extract media, ALP activity, as well as the expression of osteogenic marker genes *RUNX2a*, *OSTERIX*, *DLX5*, *OSTEOPONTIN* and *OSTEOCALCIN*, were quantified after 7 and 14 days of culture (Fig 4). With respect to ALP activity, borosilicate glass discs induced a significant decrease at both time points and in both culture media in comparison to the control (Fig 4a). Similarly, S53P4 glass in OM significantly decreased ALP activity. In case of the extracts, the undiluted extract (B1) strongly inhibited ALP activity regardless of the time point or the presence of osteogenic supplements (Fig 4g). However, the diluted extracts had no effect on ALP activity. Regarding the osteogenic marker genes, upon disc culture the expression of *RUNX2a* was stimulated by S53P4 but not by the borosilicate glasses (Fig 4b). In extract culture, B1 and B2 extracts moderately increased the expression of *RUNX2a* (Fig 4h). *OSTERIX* and *DLX5* expression was stimulated by all the glasses in disc culture but the effect was most pronounced with B25 and B50 glasses (Fig 4c and 4d). *OSTEOPONTIN* expression, on the other hand, was induced only by the borosilicate glasses, out of which B25 induced a stronger response (Fig 4e). *OSTEOCALCIN* expression was not regulated by any of the glass discs (Fig 4f). In the extract culture, there was an elevation in *OSTERIX*, *DLX5* and *OSTEOPONTIN* expression induced by the undiluted extract (B1) (Fig 4i, 4j and 4k), whereas the diluted extracts had no clear effect. Similar to the disc culture, *OSTEOCALCIN* expression did not differ from the controls in either of the extract media (Fig 4l).

### Collagen-I production in BaG disc and extract cultures

**Fig 5:**
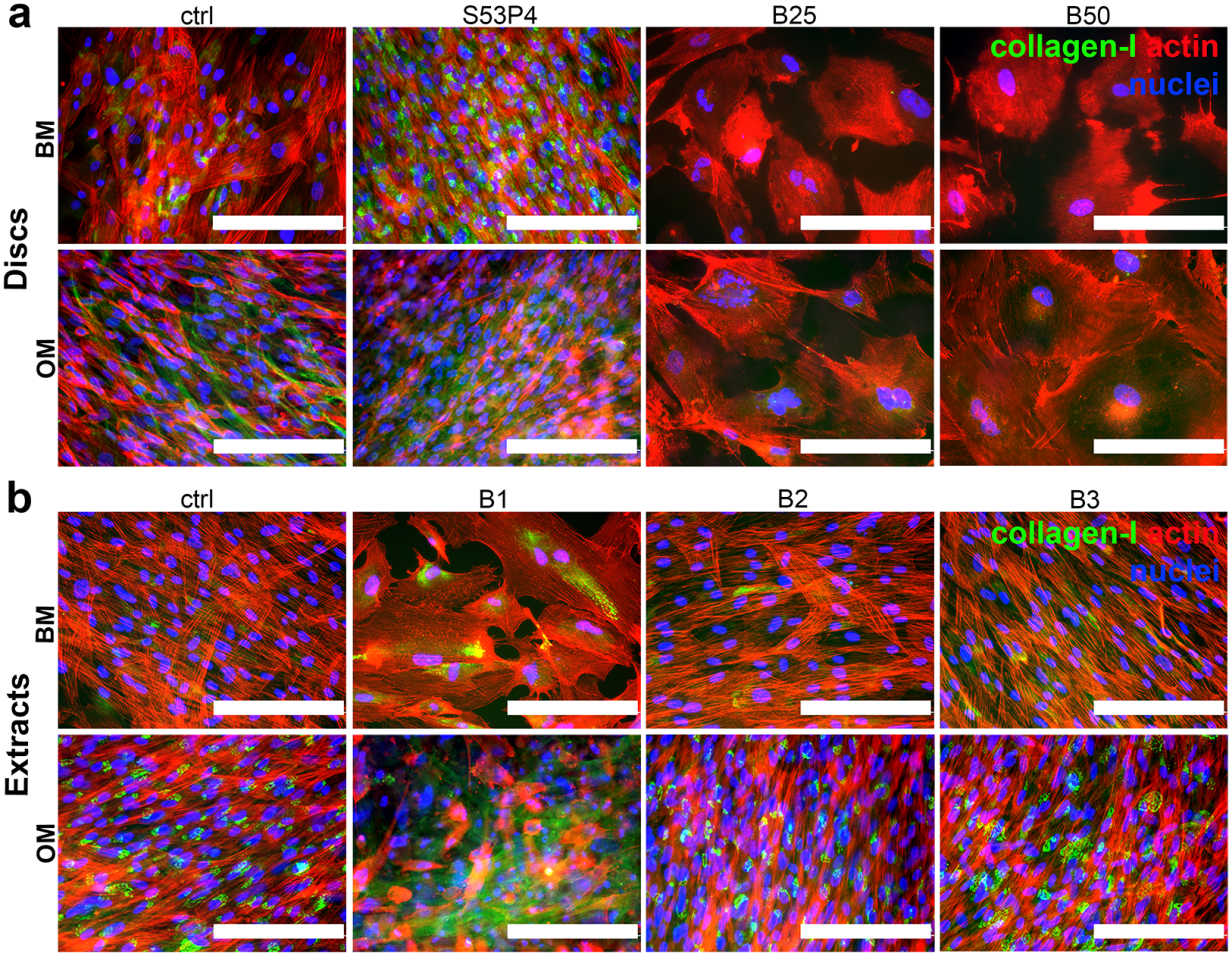
Collagen-I production in response to borosilicate glasses. **a**. Immunocytochemical staining of collagen-1 (green) in hASCs cultured on glass discs for 21d. **b**. Immunocytochemical staining of collagen-1 (green) in hASCs cultured in borosilicate glass extracts for 18d. Nuclei were stained blue with DAPI and actin cytoskeleton red with phalloidin. Scale bars 200 μm. BM = basic medium, OM = osteogenic medium. B1 = undiluted extract, B2 = 1:10 dilution, B3 = 1:100 dilution.

Collagen-I, the most abundant protein in the organic phase of bone ECM, is produced during the osteogenic commitment of stem and progenitor cells[33], and we therefore evaluated the production of this protein in response to the BaG disc and extract cultures. As seen in Fig 5a, there is only a very low level of collagen-I production in control cells grown in BM. However, more collagen-I was detected upon culture on S53P4 discs in BM, although the location of this protein is still clearly intracellular. The secretion of collagen-I only seems to occur in OM-based cultures, both in control and S53P4 substrates. On borosilicate glass discs, on the other hand, no collagen-I could be detected in either of the media. In the extract media intracellular collagen-I production was slightly increased in B1 BM, whereas in diluted BM extracts no collagen-I production was observed (Fig 5b). Osteogenic medium clearly supported collagen-I production better than BM, but very extensive extracellular collagen-I matrix with distinct fiber-like structures was only detected in the presence of undiluted OM extract (B1).

### Late osteogenic commitment induced by BaG discs and extracts

**Fig 6:**
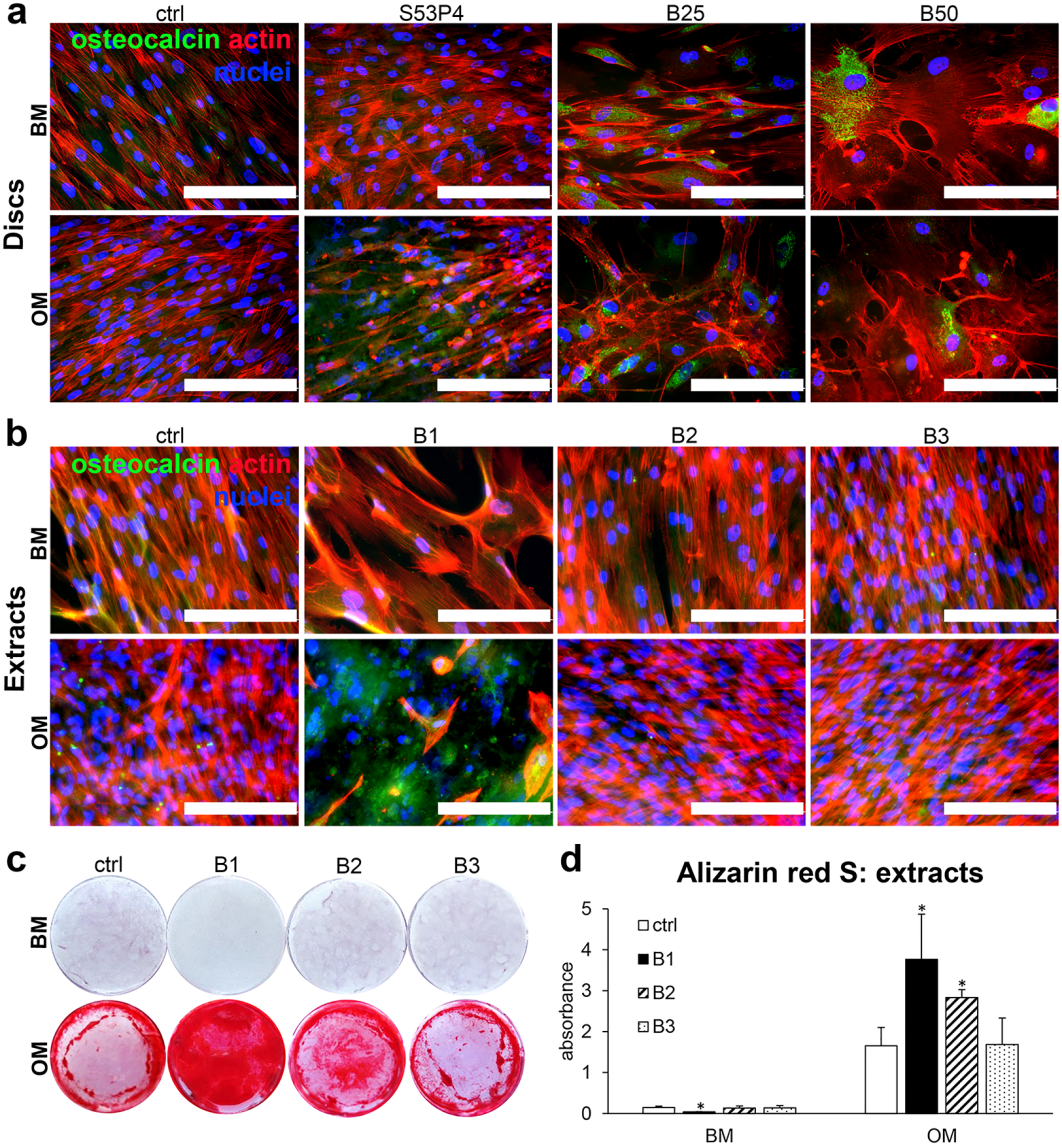
Borosilicate glass-induced late osteogenic differentiation. **a**. Immunocytochemical staining of osteocalcin (green) in hASCs cultured on glass discs for 21d. **b**. Immunocytochemical staining of osteocalcin (green) in hASCs cultured in borosilicate glass extracts for 19d. Nuclei were stained blue with DAPI and actin cytoskeleton red with phalloidin. Scale bars 200 μm. **c**. Alizarin red S staining of CaP mineral in borosilicate glass extracts after 19d of culture. Mineral is stained red. Each image represents the whole well of a 24-well plate. **d**. Quantified Alizarin red S staining at 19d. n = 6. * p<0.05 between the indicated group and the control (ctrl) group at the same time point. BM = basic medium, OM = osteogenic medium. B1 = undiluted extract, B2 = 1:10 dilution, B3 = 1:100 dilution.

The late stages of osteogenic commitment are typically characterized by mineralization of the ECM, which can be detected by different staining methods such as Ca-staining Alizarin red S. However, due to the high Ca content of the BaG discs, this method gives extensive background staining, making the detection of cell-produced mineral impossible upon direct disc culture. Instead of mineralization, we therefore analyzed the production of mineral-associated protein osteocalcin, which is equally indicative of late osteogenic commitment. Interestingly, as evidenced by Fig 6a, no osteocalcin production could be detected on either control or S53P4 substrates in either of the media, but in contrast to this, both B25 and B50 glasses upregulated osteocalcin production in both BM and OM. In the case of extracts, osteocalcin was present in none of the BM samples (Fig 6b). However, osteocalcin production was evidently upregulated in OM B1, compared to the control OM and diluted extracts, which entirely lacked osteocalcin production.

In the extract culture, with only the BaG ions present, there are no obstacles for the Alizarin red S assay, so we analyzed ECM mineralization after 19 days of culture using this staining method. As seen in Fig 6c, mineralization could not be observed in BM conditions, whereas in OM conditions excessive amount of minerals were apparent in all samples. The mineralization was most extensive in B1 and decreased upon dilution of the OM extract. When quantified, both B1 and B2 extracts significantly increased mineral production in comparison to the control in OM culture conditions (Fig 6d).

### The effect of BaG disc and extract cultures on endothelial marker expression

**Fig 7:**
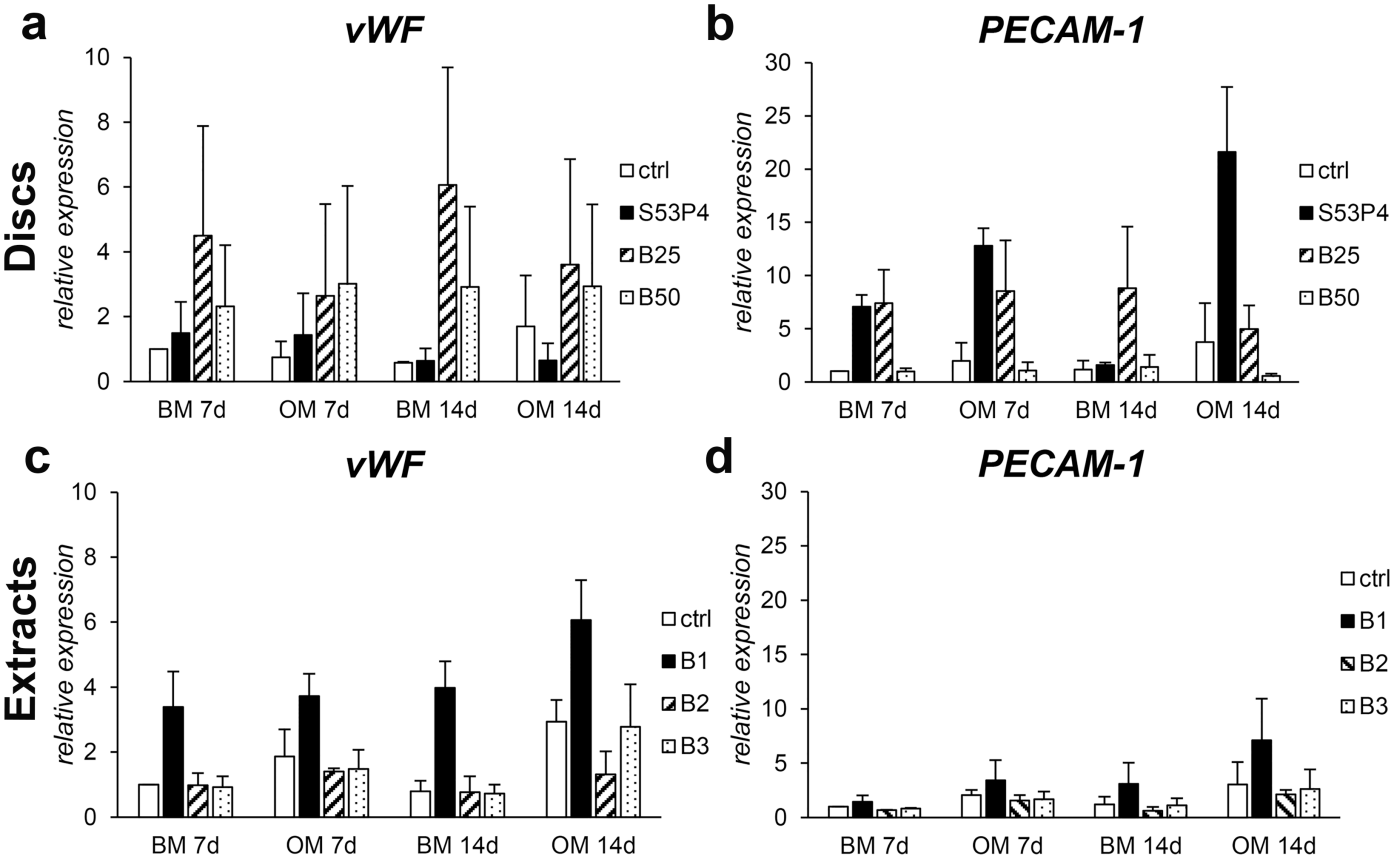
The expression of endothelial marker genes in response to borosilicate glasses. *vWF* (**a**) and *PECAM-1* (**b**) expression in hASCs cultured on glass discs. *vWF* (**c**) and *PECAM-1* (**d**) expression in hASCs cultured in borosilicate extracts. n = 2–3. BM = basic medium, OM = osteogenic medium. B1 = undiluted extract, B2 = 1:10 dilution, B3 = 1:100 dilution.

Finally, after determining the *in vitro* osteogenesis inducing effect of the borosilicate glass discs and extracts, we were interested in evaluating whether they have a role in regulating angiogenic factors. For this, we assessed the expression of endothelial marker genes *vWF* and *PECAM-1*. Indeed, borosilicate glasses had an upregulating effect on these markers (Fig 7). Regarding *vWF*, the expression was increased by both borosilicate discs (Fig 7a) as well as undiluted extract (Fig 7c) in both BM and OM. Undiluted extract also slightly stimulated the expression of *PECAM-1* (Fig 7d). In disc culture *PECAM-1* upregulation was observed upon culture on S53P4 and B25 glasses (Fig 7b).

## Discussion

Silicate BaGs have gained considerable attention since their discovery by Larry Hench in the 1960s, but their progression into widespread clinical use has been severely hampered by their slow reactivity prolonged retention in the human body. To boost the reactivity of the glasses, silicate has been totally or partially replaced with borate in 45S5 and 13–93 glasses, leading to very satisfactory degradation rates[6–9]. However, such modification has not previously been attempted with S53P4, a BaG widely used in clinical orthopedic treatments. Moreover, only little knowledge exists concerning the borosilicate glass-induced cellular responses. This prompted us to assess the behavior of hASCs in response to S53P4-based borosilicate glass compositions in direct culture, as well as in glass ion-conditioned extract media.

Initially, the glass dissolution was tested in SBF. The non-congruent dissolution of these materials led to preferential leaching of the alkaline and alkaline earth ions along with the soluble silicate. The large Ca release is assumed to lead to supersaturation of the solution and to precipitation of CaP reactive layer, which in typical bioactive glasses, such as 45S5 and S53P4, consists of hydroxycarbonate apatite[34]. With an increase in the boron content at the expense of silica an increase in both the pH and Ca release was evident. This is attributed to the increased solubility of borosilicate glasses compared to silicate glasses[7,9]. Interestingly, while the boron concentration in the solution rose with increasing boron content in the glass, the silica release remained constant, regardless of the Si/B ratio, indicating a preferential leaching of the borate phase of the glass as reported in[35]. The concentration of P decreased over the immersion period in the case of all the glasses, which is typical of the precipitation of a CaP reactive layer[36]. The precipitation of a reactive layer was further confirmed using EDX/SEM, showing that all the glasses exhibited the formation of a Si-rich layer and, successively, a CaP layer. Moreover, with an increase in the glass’ boron content, the thickness of these reactive layers increased. However, regarding the immersion testing, it should be kept in mind that the SBF testing conditions differ significantly from those present in the cell culture setup. Moreover, it has been demonstrated that SBF may not be the optimal testing medium since this solution is already supersaturated towards the precipitation of HA[37]. We therefore evaluated the reaction layer formation also in cell culture conditions, i.e. upon immersion of the discs for 21 days in cell culture medium. In these conditions, a clear formation of the reaction layers at the surface of the discs was also detected. Moreover, similarly to in SBF, the thickness of the reactive layers increased with increasing glass boron content, suggesting a similar dissolution behavior between these two immersion conditions.

As indicated by the live/dead staining and quantitative proliferation assay, the cell amount clearly decreased upon culture on borosilicate glass discs, as well as in undiluted extract media, suggesting that the modified glasses have an inhibitory effect on cell proliferation. In fact, similar results have been obtained with borosilicate glasses based on 45S5 and 13–93 compositions[7,15,18]. It is known that boron, when supplemented in the culture medium in the form of boric acid, significantly inhibits the proliferation of hBMSCs, as well as MC3T3-E1 cells, at concentrations higher than 1,000 ng/mL (≈0.09 mM)[13,14]. Moreover, Brown et al. observed a 50% reduction in cell density with boron ion concentration of 2.5 mM[7]. However, in case of BaG culture, cells are simultaneously exposed to a mixture of different ions and it is thus not straightforward to specify the effect of boron alone. Indeed, due to their higher reactivity, borosilicate glasses release increased amounts of other ions as well, out of which at least Ca has been previously associated with inhibitory effect on cell proliferation[38]. Moreover, increased reactivity is accompanied with an increase in the pH of the culture medium, which can also negatively affect cells’ proliferative capacity and thus partially explain the reduced cell numbers on borosilicate glasses[39]. Therefore, the decreased proliferation on borosilicate BaGs might not be solely due to boron. Indeed, Fu and co-workers demonstrated with 13–93 based extract medium that cell proliferation is negatively affected by boron concentrations over 0.65 mM[16], a significantly higher value than the 0.09 mM determined for boron alone. This is similar to our observations; B2 extract with 0.5 mM boron did not compromise cell proliferation, suggesting that cells might tolerate higher boron concentrations when boron is part of the glass dissolution ion mixture.

One approach to limit the negative effect of ionic release from borosilicate BaGs on cell proliferation is to conduct cell culture in dynamic fluid flow conditions rather than as a static culture, as proposed by Brown and co-workers[7]. This *in vivo*-mimicking approach prevents the increase of ion concentrations and pH to toxic levels, thus potentially improving cell survival. However, even the dynamic culture could not entirely resolve the problem related to the decreased proliferation[7]. Moreover, the lack of proliferative capacity does not necessarily correlate with decreased differentiation ability. In fact, it is known that osteoblastic differentiation is accompanied by reduction in cell amount, which is necessary for the maintenance of the tissue organization[19].

Upon culture in both direct contact with the borosilicate glasses, as well as in the extract media, we detected a distinct change in cell morphology, which seemed to become even more pronounced with increasing borate content in the glass. The cells adopted a rounded appearance with large surface area, as opposed to the elongated spindle-shaped cells on polystyrene and S53P4. Similar boron-associated cell enlargement was reported in the study of Wang and coworkers who cultured rat BMSCs on boron nitride nanotube-coated surfaces[40]. It is known that active osteoblasts adopt a spread and cuboidal shape and associate tightly to each other[41,42], supporting the hypothesis that osteoblastic induction of hASCs may occur in response to borosilicate glasses.

To determine the osteogenic differentiation of hASCs in response to borosilicate glasses, we analyzed the ALP activity, expression of osteogenic marker genes, production of collagen-I and osteocalcin proteins and, in case of the extract media, mineralization. Even though boron supplementation has previously been shown to induce ALP activity of hBMSCs[13], in the present study, ALP activity was significantly downregulated by both direct culture and borosilicate extract media. In fact, 13-93-based borosilicate glasses have also been linked with a reduction in ALP activity[15–17]. However, the negative effect of borosilicate BaGs on ALP activity might not be necessarily related to boron, since also calcium, released in high concentrations from fast-reacting borosilicate BaGs, has been shown to downregulate ALP activity[43–46]. Moreover, ALP activity is not considered a very reliable indicator of osteogenic commitment [20,21] and even with reduced ALP activity levels cells can differentiate efficiently towards osteoblastic phenotype, as demonstrated in our previous study[22]. Indeed, in gene expression level, both direct culture on borosilicate glass discs and in undiluted extract media increased the expression of osteogenic marker genes *OSTERIX*, *DLX5* and *OSTEOPONTIN* in both BM and OM culture conditions. Furthermore, we saw an upregulation of collagen-I production in undiluted extract media, and regarding the later stages of osteogenesis, the production of mineral-associated protein osteocalcin was stimulated by borosilicate glass discs in both BM and OM, and by undiluted extract in OM. In the case of the extracts, significant concentration-dependent induction of mineralization was observed in the OM-based cultures, in comparison to the control OM. Overall, this data strongly suggests that, despite the attenuation of proliferation and ALP activity, S53P4-based borosilicate BaGs induce the commitment of hASCs towards osteogenic fate. Interestingly, in direct culture on discs this is evidenced in both BM and OM, whereas in extract media the OM supplements are needed for full commitment. This fits well in line with our previous study with silicate BaG extracts [22] and suggests that cell attachment on the glass surface is an important factor regulating cell behavior. In fact, in case of S53P4 and 1–06 glass compositions, we recently demonstrated that the early osteogenesis of hASCs is regulated by attachment-related mechanisms and does not occur in the presence of glass ions alone[47].

Interestingly, in addition to the osteogenic markers, borosilicate glasses were also observed to upregulate the expression of two endothelial marker genes, *vWF* and *PECAM-1*. Silicate BaGs are well known for their ability to stimulate the secretion of angiogenic growth factors and induce tubule formation *in vitro*, as well as vascularization *in vivo*[48–53]. It was also reported that the ionic dissolution products from a 45S5-based borosilicate glass can stimulate HUVEC tubule formation and pro-angiogenic cytokine secretion[27], which supports our observations. In contrast to this, *in vivo* 13–93 based borosilicate glasses were actually observed to stimulate less vessel formation in rat critical-sized calvarial bone defects, in comparison to silicate 13–93[23], suggesting a negative effect of borosilicate glasses on vascularization. However, this *in vivo* result cannot be directly compared to *in vitro* observations, especially when the glass composition is different. It is thus possible that the S53P4- and 45S5-based borosilicate glasses support vessel formation, but clearly more *in vitro* and *in vivo* evaluation is needed to support this statement.

## Conclusions

We were able to demonstrate that S53P4-based borosilicate glasses have favorable effects on hASC osteogenic commitment. In fact, in the disc culture the borosilicate BaGs even outperformed the unmodified S53P4 in the cases of some of the osteogenic markers analyzed, further highlighting their potential to alleviate the problems associated with slow-reacting silicate BaGs. However, despite the good differentiation response, some concerns are raised by the strong inhibitory effect of borosilicate glasses on cell proliferation. Still, it is good to keep in mind that *in vivo* the conditions are more dynamic and the problem of locally excessive ionic concentrations and pH are presumably diminished. Moreover, in addition to silicate replacement with borate, the properties of BaGs are possible to further tune with countless other chemical modifications. Thus, this toolkit should be more extensively exploited to adjust the BaG properties to the desired direction and, ultimately, to be able to introduce a glass with optimal clinical performance.

## Supporting information

S1 Table Raw data. Numerical data underlying the findings of the study.
(XLSX)
